# New Casemix Classification as an Alternative Method for Budget Allocation in Thai Oral Healthcare Service: A Pilot Study

**DOI:** 10.1155/2010/231398

**Published:** 2010-09-20

**Authors:** Thunthita Wisaijohn, Atiphan Pimkhaokham, Phenkhae Lapying, Chumpot Itthichaisri, Supasit Pannarunothai, Isao Igarashi, Koichi Kawabuchi

**Affiliations:** ^1^Healthcare Economics, Graduate School of Medical and Dental Sciences, Tokyo Medical and Dental University, Tokyo 113-8510, Japan; ^2^Department of Oral and Maxillofacial Surgery, Faculty of Dentistry, Chulalongkorn University, Bangkok 10330, Thailand; ^3^Dental Health Bureau, Department of Health, Ministry of Public Health, Nonthaburi 11000, Thailand; ^4^Faculty of Medicine, Naresuan University, Phitsanulok 65000, Thailand

## Abstract

This study aimed to develop a new casemix classification system as an alternative method for the budget allocation of oral healthcare service (OHCS). Initially, the International Statistical of Diseases and Related Health Problem, 10th revision, Thai Modification (ICD-10-TM) related to OHCS was used for developing the software “Grouper”. This model was designed to allow the translation of dental procedures into eight-digit codes. Multiple regression analysis was used to analyze the relationship between the factors used for developing the model and the resource consumption. Furthermore, the coefficient of variance, reduction in variance, and relative weight (RW) were applied to test the validity. The results demonstrated that 1,624 OHCS classifications, according to the diagnoses and the procedures performed, showed high homogeneity within groups and heterogeneity between groups. Moreover, the RW of the OHCS could be used to predict and control the production costs. In conclusion, this new OHCS casemix classification has a potential use in a global decision making.

## 1. Introduction

There are many insurance systems worldwide for Universal Healthcare Coverage. In Thailand, health insurance systems are categorized into three major schemes: the Civil Servant Medical Benefit Scheme (CSMBS), the Social Security Scheme (SSS), and the Universal Coverage Scheme (UCS) or the “30 baht (in 2002, 43.0 Baht/US$ copayment) for all diseases” (UCS was implemented in May 2001 and introduced nationwide in April 2002) [1]. In 2006, the UCS abolished the 30 baht copayment per visit and made the UCS free [2]. In the past, the health budget was allocated by the characteristics of each healthcare provider, the number of doctors, and the number of patient beds. Thus, healthcare resources were not equitably allocated between the health insurance systems [3]. In 2001, the revamping of the health insurance system was initiated to restructure the methodology and the system allocating healthcare resources by the Health Systems Research Institute [4, 5]. One key difference between the insurance schemes is that the UCS separated the provider budget between the inpatient and the outpatient for exclusive capitation. Under this paradigm, the outpatient budget was allocated on the basis of the capitation rate, while the inpatient budget was allocated on the basis of Diagnosis Related Group (DRG) within a global budget [1]. 

Under the UCS, the budget for the oral healthcare service (OHCS), based on the benefit package, is part of the outpatient and Promotion/Prevention budget, to share financial risks among OHCS and general health services. While the budgets for all capital investment budgets are allocated by the Ministry of Public Health (MOPH) regional regulators' judgment, the OHCS, unfortunately, is usually the last priority. Therefore, the efficiency of the allocation is doubted, mainly the methodology and reliability aspect, as follows: (1) Since the Universal Healthcare Coverage policy was established in the year 2001, the demands for government-funded OHCS have increased significantly. In particular, the demand for dental substitution, which is highly expensive, has increased [6]. The per capita budget for all healthcare service in the fiscal year of 2003 was the same amount as the previous year, approximately 1,202 baht (43.0 Baht/US$) [7, 8] and became 2,202 Baht (34.34 Baht/US$) in 2009. (2) Current budget allocation has not been categorized for OHCS separately, and there have been few cost studies of OHCS. The information on the cost of these service activities is scarce, leading to a shortage of information for making management decisions. Thus, cost studies of OHCS are important and necessary for the evaluation of healthcare managerial efficiency and resource allocation, as well as for generating the appropriate parameters to use in making policies for healthcare service improvement in the context of budgetary constraints [9, 10].

Casemix is a generic term for the patients' classification system, including inpatient and outpatient status, budgeting allocation, and payment [11]. Successful outcomes from the adoption of a casemix system have been shown in many countries [12–14]. The best-known classification system used in a casemix funding model is the DRG. The DRG classifies acute inpatient episodes into a discrete number of manageable categories, depending on their clinical condition and resource consumption, assigned by a grouper program based on their demographics, clinical information (diagnosis (Dx) and procedure (Proc) codes), and comorbidity. The DRG method has been well evaluated for classifying inpatient treatment [15, 16]. In Thailand, the initial DRG was implemented in 1999 and several successive versions have been developed. Pannarunothai's studies on DRG development in Thailand recommended that casemix systems should be used in the budget allocation for the healthcare service system [11, 17].

In Thailand, most people are covered by the aforementioned insurance schemes, although the budget allocations and payment systems are different between the schemes. DRG is currently employed as an allocation method only for inpatient healthcare budgets and not for all healthcare budgets. However, the DRG system has limitations in reflecting the OHCS cost. The future development of the appropriate inpatient and outpatient casemix for OHCS is important and necessary for economic healthcare management [17].

For the above-described reasons, the development of a new casemix classification in OHCS is desirable. This study aimed to develop and examine the feasibility of a new casemix classification system as an alternative method for budget allocation in Thai OHCS. These three schemes were each adapted, on the basis of DRG, as an alternative method to inpatient and outpatient-related OHCS for budget allocation [11, 18, 19]. This system might, in time, be applied in other countries healthcare system for OHCS resource allocation as well.

## 2. Materials and Methods

This study was conducted utilizing the electronic data of individual patients treated from April 2008 to March 2009 at three selected tertiary hospitals that met the study's inclusion criteria. The study protocol was approved by the Ethics Committee of the participating hospitals. The inclusion criteria were the use of the International Classification of Diseases, 10th edition (ICD-10) and International Classification of Disease, 9th edition, Clinical Modification (ICD-9-CM) for the clinical records [20, 21] and the systematic keeping of a database record that was based on a global coding of the clinical records. These databases contained information on inpatient and outpatient care utilization, including demographic data (date of birth (DOB), age, and gender), clinical information (Dx, Proc), and resource consumption (hospital charge, admission date, discharge date and type, length of stay (LOS), and health insurance). The five main methods used to develop a new casemix classification as an alternative method for budget allocation of OHCS in this study were coding, classification, costing, calibration, and payment. 

### 2.1. Coding

The coding process was divided into two parts.

**Part I**

The development of the new casemix classification for OHCS began with the adoption of the ICD-10, ICD-9-CM, Current Dental Terminology 2007 (CDT) [22], International Statistical of Diseases and Related Health Problem, 10th revision, Thai Modification (ICD-10-TM) [23, 24], Thai DRG version 4 [25], and International Refined DRG (IR-DRG) [26]. The study designed the analysis method in two steps. *Step one*, the ICD-10-TM for Dx and Proc codes related to OHCS were retrieved by a researcher and approved by five dentists with more than ten years of clinical experience and specialists in OHCS. *Step two*, these codes were then mapped to ICD-10 for Dx and ICD-9-CM for Proc by Program Map version 1.0, copyright of Thai health coding center, Cluster for Health Information Division, Bureau of Policy and Strategy, MOPH, Thailand. These selected codes were used as inclusion lists of principal diagnoses (PDx) and procedures (Proc).

**Part II**

**Figure 1:**
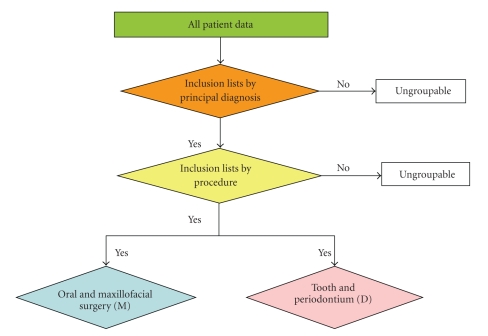
Diagram presents the steps one and two.

The electronic data of individual patients from the three selected tertiary hospitals were checked based on the inclusion lists of PDx and Proc (Figure 1).

### 2.2. Classification

To develop a new casemix classification system for Thai OHCS, a specially designed computer software program called “Grouper” was used. Grouper was able to allocate each episode to a DRG according to the clinical information and other relevant data. This program used clinical and demographic data as the input and produced a corresponding DRG as the output [27].

**Figure 2:**
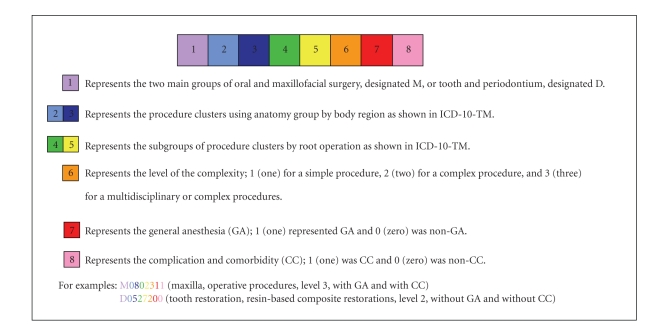
Diagram presents the casemix classification model of OHCS (one procedure in one visit) as represented in eight-digit codes.

**Figure 3:**
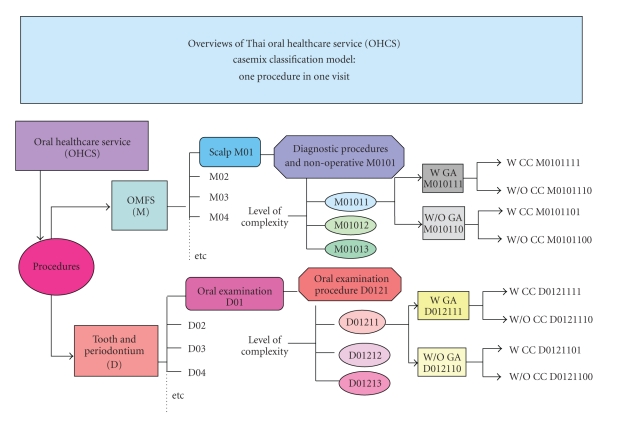
Diagram demonstrates the overviews of the Thai oral healthcare service (OHCS) casemix classification model of one procedure in one visit. M = oral and maxillofacial surgery (OMFS), W = with, W/O = without, GA = general anesthesia, CC = complication and comorbidity.

**Figure 4:**
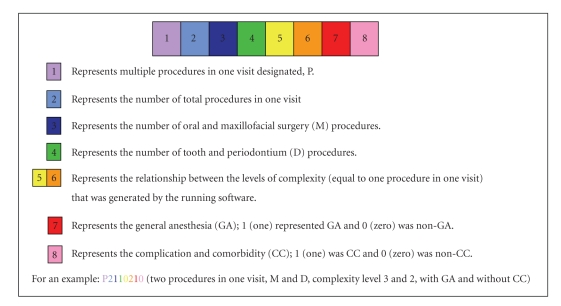
Diagram presents the casemix classification model of OHCS (multiple procedures in one visit) as represented in eight-digit codes.

**Figure 5:**
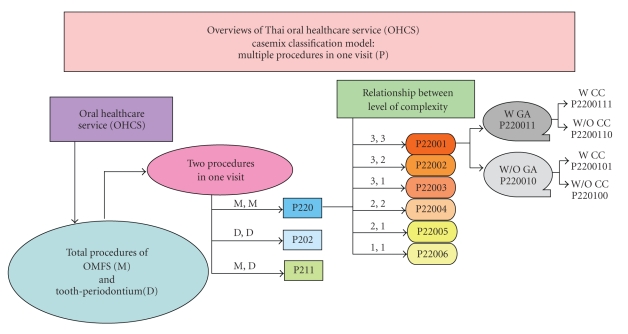
Diagram demonstrates overviews of the Thai oral healthcare service (OHCS) casemix classification model of multiple procedures in one visit (P). M = oral and maxillofacial surgery (OMFS), W = with, W/O = without, GA = general anesthesia, CC = complication and comorbidity.

The OHCS casemix classification model (Grouper) consisted of one procedure in one visit that classified cases into two main groups: the oral and maxillofacial surgery (OMFS) group, designated M, and the tooth and periodontium group, designated D. Multiple procedures in one visit were designated as P. The variables used for the OHCS grouping were included (1) PDx, (2) secondary diagnosis (SDx), (3) Proc (which were classified by the level of complexity by the same expert group), (4) anatomy group by body regions related to ICD-10-TM (Table 1), (5) root operation related to ICD-10-TM (Table 2), (6) general anesthesia (GA), and (7) complication and comorbidity (CC), using the Charlson index. This classification system was developed to allow the translation of dental procedures into eight-digit codes as summarized in Figures 2, 3, 4, and 5.

### 2.3. Costs

Initially, multiple regression analysis was used to study the relationship between the factors used for developing the codes and resource consumption. This analysis was employed to explore the relationship between the cost of P, D, and M (dependent variables) and several independent variables including, GA, CC, number of procedures in one visit, Proc (separated by the level of complexity), and LOS. Cross-validation was used to test the validation of the casemix in a separate set of data. Cross-validation showed the quality of the prediction equation between the structure data and the data for validation. The higher confidence obtained from the cross-validation, the more suitable the estimation of the population prediction equation.

### 2.4. Calibration

Three statistical analyses, the coefficient of variation (CV), the reduction in variance (RIV), and the relative weight (RW), were applied to verify the minimum variation within each group, the maximum variation among groups, and the assignment of a payment weight for the new casemix classification, respectively.

The CV is calculated as the standard deviation divided by the arithmetic mean. The CV value demonstrates the homogeneity of the cases within each group. A high CV indicates wide variation within each group. The accepted standard for CV is that each class should have a CV of less than 1.0 [15]. The expected end results using the new grouper program are groups of cases that are clinically similar and/or homogeneous with respect to resource use.

The RIV statistic is commonly used to assess the overall performance of the grouping method by comparing the variances of cost before and after grouping. The RIV was also related to the amount of variation within the data that requires explanation. A higher RIV reflected better performance of the grouping. 

The RW is a measure of the resources used: it compares the average resource used in each group with the average resource used in all cases. In this study, statistical outliers beyond three standard deviations of the average cost for each OHCS classification were eliminated [28–30]. The RW was computed based on the cost data. It was defined in our study as the mean cost in each group divided by the mean costs of all patients. The cost in this study focused only on the cost of surgery, including general and local anesthesia, medical devices and instruments, and medical supplies. The staff cost was not included in this study because it was not paid on a per case basis. In Thailand, all staff salary is paid by the government and is dependent on the degree of education and the years of experience. Furthermore, the standard of staff cost has not been well established in Thailand.

### 2.5. Payment

A payment calculation was necessary to establish the prospective payment system. The payment was calculated using the RW of the OHCS classification multiplied by the current reimbursement rate (average base rate) in each group.

## 3. Results

### 3.1. Coding

**Part I (Steps One and Two)**

The ICD-10-TM, consisting of 813 diagnoses and 1,090 procedures related to OHCS, were retrieved and mapped to ICD-10 and ICD-9-CM, respectively.

**Part II**

The electronic data of individual patients from three selected tertiary hospitals were checked by an inclusion list of PDx and Proc. There were 16,165 (84.64%) cases out of 19,098 initial cases (cases with incomplete data were eliminated) that met the inclusion criteria. The number of inpatient and outpatient cases were 2,709 (16.8%) and 13,456 (83.2%), respectively. The demographic details, clinical information and health insurance showed that the majority of the patients were female (8,723 cases; 54.0%), non-GA (13,911 cases; 86.05%), non-CC (15,708 cases; 97.17%), and CSMBS (6,368 cases; 39.4%).

### 3.2. Classification

The new OHCS casemix classification model (Grouper) consisted of two major procedure categories, M and D, 62 procedure clusters (Table 1), 165 subgroups of procedure clusters (Table 2), and 1,624 OHCS classifications according to the treatment procedures (Annex Table 5). Each OHCS classification described a cluster of patients with related diagnoses, requiring a similar examination and incurring similar treatment costs. There were 16,165 patients who were grouped into OHCS classifications by the grouper software. After the grouping process, only 307 OHCS classifications were achieved to cover these procedure codes. This result was likely limited by the OHCS data, as the amount available in this pilot study was not sufficient to support the OHCS grouper.

### 3.3. Costs

For predicting costs, regression analysis was employed. Table 3 presents the determination of the cost of P, D, and M. Because cost did not present a normal distribution, a normal logarithmic transformation was undertaken. The predicted cost of P, D, and M had R^2^values of 0.892, 0.132, and 0.122, respectively, and the probability of the F-test statistic was 0.000. The results showed that the GA, CC, number of procedures in one visit, Proc (divided by the level of complexity), and LOS were associated with the costs of P, D, and M.

### 3.4. Calibration

To ensure that the OHCS classifications reflected resource homogeneity within groups and heterogeneity between groups, the CV and RIV, respectively, were used for analysis.

The lowest CVs relative to the outpatient groups for P, D, and M were 0.02 (P2110200), 0.01 (D0843200), and 0.19 (M4004100), respectively, while the highest CVs relative to these groups for P, D, and M were 0.87 (P2020500), 0.99 (D0736200), and 0.83 (M4310101), respectively. The lowest CVs relative to the inpatient groups for P, D, and M were 0.31 (P2200511), 0 (the number of cases was less than five), and 0.22 (M4004100), respectively, while the inpatients' highest CVs for P, D, and M were 0.98 (P2200410), 0 (the number of cases was less than five), and 0.99 (M3002110), respectively. Moreover, all OHCS classifications had a CV on cost of less than one (Table 4, Annex Table 5).

The RIVs relative to the outpatient groups for P, D, and M were 27, 87, and 65 %, respectively, while the inpatient groups' RIVs for P, D, and M were 16, 0 (number of cases less than five) and 22 %, respectively. The results showed that 100 % of the OHCS classifications had a higher RIV (RIV greater than 0) on cost (Table 4). Both the CV and RIV analysis demonstrated the superior performance of the grouper software.

The lowest RWs in the outpatient groups for P, D, and M were 0.51 (P3121800), 0.14 (D0222100), and 0.30 (M1999101), respectively, while the highest RWs of the outpatient groups were 3.59 (P3121000), 21.33 (D0843200), and 7.88 (M3002200), respectively. The lowest RWs relative to the inpatient groups for P, D, and M were 0.13 (P2200600), 0 (the number of cases was less than five) and 0.02 (M1999100), respectively, while the inpatients' highest RWs were 2.03 (P3300510), 0 (the number of cases was less than five), and 3.46 (M3202210), respectively (Table 4, Annex Table 5). A high RW indicated a higher case complexity and more resources required for treatment than for low RW cases. Moreover, RW was the most important result of the calibration because it was the determinant for the payment to healthcare providers.

### 3.5. Payment

This study calculated the base rate characteristics by splitting cases into three main treatment groups consisting of P, D, and M and two patient groups consisting of inpatient and outpatient. The results showed that the highest base rate among the three main treatment groups relative to the outpatient groups and the inpatient groups were D and P, respectively.

## 4. Discussion

This is a study of the preliminary development of a new casemix classification system related to ICD-10-TM in Thai OHCS. The results indicated that the new casemix system was reliable and could possibly be utilized as a first version. However, many points need to be discussed.

### 4.1. Coding

In Thailand, the healthcare system has implemented ICD-10 for Dx and ICD-9-CM for Proc for many years. Recently, ICD-10-TM has been implemented to provide more details in the OHCS coding by modifying it with International Classification of Disease to Dentistry and Stomatology (ICD-DA) for Dx and CDT for Proc. The most recent modification of the codes was finished in 2003 [23, 24]. Thus, the ICD-10-TM provides an advantage in terms of accuracy and comprehensive coverage for Dx and Proc. It is unique, but similar to the CDT and matched with Proc one by one while ICD-9-CM has many Proc for each code. These differences indicate that ICD-10-TM was suitable, although challenging, for this study.

### 4.2. Classification

The development of the new casemix classification for OHCS concentrated on practical application of the IR-DRG that was instituted by 3M Health Information System. IR-DRG was able to classify inpatient and outpatient status and was also useful for appraising the potential for replacement on both short stay and ambulatory treatments [31]. Taking this fact into consideration, the new casemix classification was classified by the nature of the patients' procedures rather than by their diagnoses. This classification system was developed to allow the translation of dental procedures into eight-digit codes using various variables to obtain a homogenous resource group.

### 4.3. Costs

There are a variety of methods for estimating the provider's cost. However, each method has limitations. The cost of OHCS varies depending upon several factors: the characteristics of the OHCS, the scope and complexity of the treatment, the specialty and experience of physician, the high investment cost, and the location of the practice. It was difficult to measure the total submitted charges for cases in each OHCS classification. Therefore, the cost in this study was calculated based on the use of the patient payment to estimate the provider's cost.

It was expected that this method would be suitable to calibrate the OHCS classification in this study. However, a future study using a resource-based relative value scale (RBRVS) would be recommended. Because RBRVS is a schema that is used to calculate what medical providers should be paid, it assigns a relative value unit (RVUs) to each physician service that is based on three items: the physician, the practice expense, and the malpractice expense. This schema is currently used by Medicare in the United States and by nearly all Health Maintenance Organizations (HMOs) [32–34]. In 2009, Relative Value Studies Incorporated (RVSI) of Denver, Colorado developed Relative Values for Dentists (RVD), a RBRVS for dentistry that is currently indexed to the Current Dental Terminology (CDT) and supplemented by additional coding as recommended by practicing dentists [35].

### 4.4. Calibration and Payment

Because of the wide range of costs among the set of P, D, M, inpatient, and outpatient categories, the weight should reflect the relative cost of providing care and the health resources required in each DRG. A CV of less than one and a higher RIV would be expected in the new OHCS classification. Thus, the RIV, RW, and base rate characteristics were split into three main treatment groups consisting of P, D, and M and two patient groups consisting of inpatient and outpatient for the following reasons.

- The higher RIV reflects the better performance of the grouping. However, P had the lowest RIV in both inpatient and outpatient groups because P included multiple procedures in one visit, and in some cases P had M and D in one visit. P also had more data and variation than did the other groups. The RIV of M of the inpatient group was lower than the RIV of M of the outpatient group because all inpatient procedures were major surgeries with GA, complex, and costly procedures. D had the highest RIV in the outpatient group because all procedures in the outpatient group were minor surgeries, tooth and periodontium treatment, without GA.
- The procedures of each group were different according to the anatomy group, root operation, and total procedures in one visit.
- Elementary procedures in the OHCS were wildly different in each group. The OHCS was mostly focused on handiwork. OHCS was intensively skilled, time-consuming labor with a high investment cost for the equipment, instruments, and materials [36].

Taken together, this new OHCS grouper has been potentially implemented in Thailand. However, in some countries that use ICD-10 for Dx, ICD-9-CM for Proc and DRG for budget allocation, this information of the mapping process could be used as a guideline to further develop their own systems. Moreover, the benefits of the DRG grouper for OHCS could be used for expenditure estimation, resource allocation, payment, and oral healthcare finance focus.

### 4.5. Limitation

The problems related to the implementation of a new casemix classification for OHCS are elaborated as follows.

- Although computerized information systems are widely used in Thailand, only a few hospitals provided good clinical data that were ready to use [11]. This was an important issue for OHCS information because ICD-10 and ICD-9-CM were not commonly used for recording with the same standardization. This problem was likely due to the limited data from OHCS in Thailand, which could not support an OHCS grouper.
- The diagnoses and procedures of the OHCS coding system are scattered throughout the ICD-10 and ICD-9-CM, making them difficult to find and use. Moreover, currently OHCS codings are not being used effectively in dentistry.
- The OHCS patient data from this study did not involve patients from university hospitals because the patients in university hospitals had more heterogeneity in their diagnoses and services than did other hospitals. In addition, OHCS classification subgroups may be needed to more precisely describe the resource consumption in the university hospital setting.
- There were 1,624 OHCS classifications. In this pilot study, the data were limited and were not sufficient to support the OHCS grouper. A large number of OHCS classifications might be difficult to handle as a good payment tool. In the future, the OHCS classification should decrease the number of groups to provide more efficiency and effectiveness in payment.
- OHCS cost weights were calculated using only cost data from some areas that might not be applicable to all hospitals in Thailand. This indicated that an expanded number of cases and data from additional hospitals would give a more exact cost weight.

## 5. Conclusion

This study demonstrated the validity of the new OHCS classification, showing high homogeneity of the cases within each group and heterogeneity of the cases between each group. Furthermore, it could be used to predict and control production costs. Therefore, this OHCS casemix classification has the potential to be used in global decision-making in the future. Moreover, some countries using ICD-10 for diagnoses, ICD-9-CM for procedures, and DRG grouper for budget allocation might be able to apply this mapping process as a guideline to develop their own system, which might benefit from the use of the DRG grouper for expenditure estimation, resource allocation, payment, and healthcare finance focus.

## Figures and Tables

**Table 1:** Examples of both groups (M and D), split into procedure clusters using the anatomy group by body region.

OMFS groups (anatomical body region)	Code M
Scalp	
*Include:* scalp and subgaleal soft tissues	M01
Skull	M02
Cranial nerves X, Trigeminal nerve	M03
Cranial nerves X1, Accessory nerve	M04
Cranial nerve XII, Hypoglossal nerve	M05
Cranial nerves	M06
Face	M07
Maxilla	M08
Mandible	M09

*Tooth and periodontium group (anatomical body region)*	*Code D*

Oral examination	D01
Radiographs/Diagnostic imaging	D02
Preventive dentistry	D03
Oral hygiene instructions and counseling	D04
Tooth restoration	D05
Endodontic treatment	D06

M: oral and maxillofacial surgery (OMFS) corrects a wide spectrum of diseases, injuries, and defects in the head, neck, face, jaws, and the hard and soft tissues of the oral and maxillofacial region.

D: tooth and periodontium: periodontium refers to the specialized tissues that surround and support the teeth (small, calcified, whitish structures found in the jaws (or mouth)) maintaining them in the maxilla and mandible.

**Table 2:** Examples of the procedure clusters, split into subgroups of procedure clusters using a root operation.

OMFS groups (root operation)	Code M
*Scalp*	
*Include:* scalp and subgaleal soft tissues	*M01*
Diagnostic procedures and non-operative procedures	M0101
Operative procedures	M0102
Miscellaneous procedures	M0104
Other procedures and operations	M0199

*Tooth and periodontium (root operation)*	*Code D*

*Oral examination*	*D01*
Oral examination procedures	D0121
*Radiographs/Diagnostic imaging*	*D02*
Intraoral film	D0222
Extraoral film	D0223
Others	D0299

M: oral and maxillofacial surgery (OMFS).

D: tooth and periodontium.

**Table 3:** Multiple regressions of the cost of multiple procedures (P), the cost of OMFS (M), and the cost of tooth and periodontium (D).

		Cost of multiple procedures (P)			Cost of OMFS (M)			Cost of tooth and periodontium (D)		
		95% CI			95% CI			95% CI		
		Odds-ratio	Lower-upper	*P*-value	Odds-ratio	Lower-upper	*P*-value	Odds-ratio	Lower-upper	*P*-value
Gender	Male (reference)									
	Female	0.97	0.89–1.13	.973	1.14	1.02–1.19	.009	0.95	0.88–1.03	.243

Age	0–22 (reference)									
	23–40	1.04	0.89–1.22	.671	1.56	1.39–1.74	<.001	1.02	0.91–1.15	.633
	41–54	0.88	0.75–1.03	.121	1.17	1.05–1.31	.04	0.82	0.73–0.92	.001
	54 +	0.85	0.73–0.99	.039	1.17	1.15–1.30	.03	0.79	0.71–0.88	<.001

GA	Non-GA (reference)									
	GA	295.78	162.41–538.67	<.001	1.57	1.49–1.66	<.001	2386.74	891.84–6387.39	<.001

Number of procedures	1 (reference)									
	2	9.55	8.72–12.45	.033	1.02	1.01–1.03	<.001	1.02	1.01–1.02	<.001
	3	2.72	1.35–4.56	<.001	1.21	1.16–1.28	<.001	1.19	1.14–1.23	<.001
	4	8.68	6.78–11.34	<.001	1.16	1.12–1.20	<.001	1.16	1.13–1.27	<.001

CC	Non-CC (reference)									
	CC	3.25	2.31–4.57	<.001	1.17	1.10–1.31	<.001	15.76	11.25–21.96	<.001

Complexity	1 (reference)									
	2-3	3.81	2.31–4.57	<.001	1.91	1.89–1.92	<.001	1.91	1.89–1.92	<.001

LOS	LE21 (reference)									
	GE22	0.15	0.06–0.33	<.001	1.53	1.38–1.73	<.001	0.02	0.05–0.08	<.001

Adjusted cost of multiple procedures (P) *R* ^2^ = 0.892, cost of OMFS (M) *R* ^2^ = 0.122, and cost of tooth and periodontium (D) *R* ^2^ = 0.132

GA = general anesthesia, Number of procedures = total procedures in one visit, CC = complication and comorbidity

Complexity = level of complexity (1 = simple procedure, 2 = complex procedure, 3 = multidisciplinary or complicated procedure)

LOS = length of stay (LE21 = less than and equal to 21, GE22 = more than 21).

**Table 4:** Summary of the statistical analysis of the coefficient of variation (CV), reduction in variance (RIV), and relative weight (RW).

OHCS	*N* (cases)		CV		RIV		RW	
	Inpatient	Outpatient	Inpatient	Outpatient	Inpatient	Outpatient	Inpatient	Outpatient
P	365	7,832	0.31–0.98	0.02–0.87	16%	27%	0.13–2.03	0.51–3.59
D	3	2,619	N/A	0.01–0.99	N/A	87%	N/A	0.14–21.33
M	2,341	3,005	0.22–0.99	0.19–0.83	22%	65%	0.02–3.46	0.30–7.88

P = multiple procedures in one visit, D = tooth and periodontium, M = oral and maxillofacial surgery (OMFS)

CV = coefficient of variation, RIV = reduction in variance, RW = relative weight, N/A = not applicable.

**Table 5:** Demonstrates examples of the oral healthcare service (OHCS) groupers.

Description of oral healthcare service (OHCS) classifications	Without GA	Without GA	With GA	With GA
	without CC	with CC	without CC	with CC
Skull, Operative procedures, level 3	M0202300	M0202301	M0202310	M0202311
Face, Operative procedures, level 1	M0702100	M0702101	M0702110	M0702111
Maxilla, Operative procedures, level 1	M0802100	M0802101	M0802110	M0802111
Maxilla, Operative procedures, level 2	M0802200	M0802201	M0802210	M0802211
Maxilla, Operative procedures, level 3	M0802300	M0802301	M0802310	M0802311
Mandible, Diagnostic procedures and nonoperative procedures, level 1	M0901100	M0901101	M0901110	M0901111
Mandible, Diagnostic procedures and nonoperative procedures, level 2	M0901200	M0901201	M0901210	M0901211
Mandible, Diagnostic procedures and nonoperative procedures, level 3	M0901300	M0901301	M0901310	M0901311
Mandible, Operative procedures, level 1	M0902100	M0902101	M0902110	M0902111
Mandible, Operative procedures, level 2	M0902200	M0902201	M0902210	M0902211
Mandible, Operative procedures, level 3	M0902300	M0902301	M0902310	M0902311
Facial bone, Operative procedures, level 2	M1002200	M1002201	M1002210	M1002211
Facial bone, Operative procedures, level 3	M1002300	M1002301	M1002310	M1002311
Nose, Repair or reconstruction, level 1	M1906100	M1906101	M1906110	M1906111
Nose, Other procedures and operations, level 1	M1999100	M1999101	M1999110	M1999111
Nasal cavity, Removal and replacement of nasal packing and Control of epistaxis level 1	M2009100	M2009101	M2009110	M2009111
Nasal cavity, Other procedures and operations, level 1	M2099100	M2099101	M2099110	M2099111
Nasal cavity, Other procedures and operations, level 2	M2099200	M2099201	M2099210	M2099211
Nasal septum, Repair or reconstruction, level 3	M2206300	M2206301	M2206310	M2206311
Frontal nasal sinuses, Repair or reconstruction, level 1	M2306100	M2306101	M2306110	M2306111
Frontal nasal sinuses, Repair or reconstruction, level 2	M2306200	M2306201	M2306210	M2306211
Frontal nasal sinuses, Repair or reconstruction, level 3	M2306300	M2306301	M2306310	M2306311
Paranasal sinuses, Incision and Excision or destruction, level 1	M2504100	M2504101	M2504110	M2504111
Parotid salivary gland, Incision and Excision or destruction, level 1	M2604100	M2604101	M2604110	M2604111
Parotid salivary gland, Incision and Excision or destruction, level 2	M2604200	M2604201	M2604210	M2604211
Salivary gland and duct, Incision and Excision or destruction, level 1	M2804100	M2804101	M2804110	M2804111
Salivary gland and duct, Incision and Excision or destruction, level 2	M2804200	M2804201	M2804210	M2804211
Neck, Diagnostic procedures and nonoperative procedures, level 1	M2901100	M2901101	M2901110	M2901111
Neck, Diagnostic procedures and nonoperative procedures, level 2	M2901200	M2901201	M2901210	M2901211
Neck skin, Diagnostic procedures and nonoperative procedures, level 1	M3001100	M3001101	M3001110	M3001111
Neck skin, Operative procedures, level 1	M3002100	M3002101	M3002110	M3002111
Neck skin, Operative procedures, level 2	M3002200	M3002201	M3002210	M3002211
Neck skin, Operative procedures, level 3	M3002300	M3002301	M3002310	M3002311
Carotid artery, Operative procedures, level 1	M3202100	M3202101	M3202110	M3202111
Carotid artery, Operative procedures, level 2	M3202200	M3202201	M3202210	M3202211
Cervical lymph nodes, Diagnostic procedures and nonoperative procedures, level 1	M3301100	M3301101	M3301110	M3301111
Cervical lymph nodes, Diagnostic procedures and nonoperative procedures, level 2	M3301200	M3301201	M3301210	M3301211
Thyroglossal reminant, Incision and Excision or destruction, level 1	M3504100	M3504101	M3504110	M3504111
Thyroglossal reminant, Incision and Excision or destruction, level 2	M3504200	M3504201	M3504210	M3504211
Lip, Incision and Excision or destruction, level 1	M3604100	M3604101	M3604110	M3604111
Lip, Incision and Excision or destruction, level 2	M3604200	M3604201	M3604210	M3604211
Lip, Repair or reconstruction, level 1	M3606100	M3606101	M3606110	M3606111
Lip, Repair or reconstruction, level 2	M3606200	M3606201	M3606210	M3606211
Floor of mouth, Incision and Excision or destruction, level 1	M3804100	M3804101	M3804110	M3804111
Mouth, Diagnostic procedures and nonoperative procedures, level 1	M3901100	M3901101	M3901110	M3901111
Mouth, Miscellaneous procedures, level 1	M3903100	M3903101	M3903110	M3903111
Mouth, Incision and Excision or destruction, level 1	M3904100	M3904101	M3904110	M3904111
Mouth, Incision and Excision or destruction, level 2	M3904200	M3904201	M3904210	M3904211
Mouth, Repair or reconstruction, level 1	M3906100	M3906101	M3906110	M3906111
Tongue, Incision and Excision or destruction, level 1	M4004100	M4004101	M4004110	M4004111
Deciduous teeth, General procedures, level 1	M4210100	M4210101	M4210110	M4210111
Permanent teeth, General procedures, level 1	M4310100	M4310101	M4310110	M4310111
Permanent teeth, General procedures, level 2	M4310200	M4310201	M4310210	M4310211
Soft palate, Repair or reconstruction, level 1	M4606100	M4606101	M4606110	M4606111
Soft palate, Repair or reconstruction, level 2	M4606200	M4606201	M4606210	M4606211
Uvula, Repair or reconstruction, level 1	M4706100	M4706101	M4706110	M4706111
Uvula, Repair or reconstruction, level 2	M4706200	M4706201	M4706210	M4706211
Uvula, Other procedures and operations on oral cavity, level 1	M4799100	M4799101	M4799110	M4799111
Uvula, Other procedures and operations on oral cavity, level 2	M4799200	M4799201	M4799210	M4799211
Uvula, Other procedures and operations on oral cavity, level 3	M4799300	M4799301	M4799310	M4799311
Oral examination, Oral examination procedures, level 1	D0121100	D0121101	D0121110	D0121111
Radiographs/Diagnostic imaging, Intraoral film, level 1	D0222100	D0222101	D0222110	D0222111
Radiographs/Diagnostic imaging, Extraoral film, level 1	D0223100	D0223101	D0223110	D0223111
Preventive Dentistry, Preventive Dentistry procedures, level 1	D0324100	D0324101	D0324110	D0324111
Tooth Restoration, Amalgam restorations (including polishing), level 1	D0526100	D0526101	D0526110	D0526111
Tooth Restoration, Resin-based composite restorations, level 1	D0527100	D0527101	D0527110	D0527111
Tooth Restoration, Resin-based composite restorations, level 2	D0527200	D0527201	D0527210	D0527211
Tooth Restoration, Crown-single restorations only, level 1	D0530100	D0530101	D0530110	D0530111
Tooth Restoration, Crown-single restorations only, level 2	D0530200	D0530201	D0530210	D0530211
Endodontic Treatment, Endodontic Treatment procedures, level 2	D0631200	D0631201	D0631210	D0631211
Endodontic Treatment, Endodontic therapy on permanent teeth, level 1	D0633100	D0633101	D0633110	D0633111
Endodontic Treatment, Endodontic therapy on permanent teeth, level 2	D0633200	D0633201	D0633210	D0633211
Endodontic Treatment, Apicoectomy/Periradicular services, level 2	D0634200	D0634201	D0634210	D0634211
Periodontal Treatment, Periodontal Treatment procedures, level 1	D0735100	D0735101	D0735110	D0735111
Periodontal Treatment, Surgical services (Including usual postoperative care), level 1	D0736100	D0736101	D0736110	D0736111
Periodontal Treatment, Surgical services (Including usual postoperative care), level 2	D0736200	D0736201	D0736210	D0736211
Prosthodontics, Prosthodontics (Removable), level 1	D0837100	D0837101	D0837110	D0837111
Prosthodontics, Prosthodontics (Removable), level 2	D0837200	D0837201	D0837210	D0837211
Prosthodontics, Fixed partial denture retainers-crown, level 2	D0838200	D0838201	D0838210	D0838211
Prosthodontics, Other fixed partial denture service, level 2	D0841200	D0841201	D0841210	D0841211
Implant services, Implant services procedures, level 2	D0943200	D0943201	D0943210	D0943211
Orthodontic treatment, Other orthodontic service, level 1	D1049100	D1049101	D1049110	D1049111
Oral health problems, Other and unspecified management of oral health problems procedures, level 1	D1251100	D1251101	D1251110	D1251111
Two procedures in one visit, M and M, complexity level 3 and level 3	P2200100	P2200101	P2200110	P2200111
Two procedures in one visit, D and D, complexity level 2 and level 2	P2020400	P2020401	P2020410	P2020411
Two procedures in one visit, D and D, complexity level 2 and level 1	P2020500	P2020501	P2020510	P2020511
Two procedures in one visit, D and D, complexity level 1 and level 1	P2020600	P2020601	P2020610	P2020611
Two procedures in one visit, M and D, complexity level 3 and level 2	P2110200	P2110201	P2110210	P2110211
Two procedures in one visit, M and D, complexity level 2 and level 2	P2110500	P2110501	P2110510	P2110511
Two procedures in one visit, M and D, complexity level 2 and level 1	P2110600	P2110601	P2110610	P2110611
Two procedures in one visit, M and D, complexity level 1 and level 2	P2110800	P2110801	P2110810	P2110811
Two procedures in one visit, M and D, complexity level 1 and level 1	P2110900	P2110901	P2110910	P2110911
Two procedures in one visit, M and M, complexity level 3 and level 3	P2200100	P2200101	P2200110	P2200111
Two procedures in one visit, M and M, complexity level 3 and level 2	P2200200	P2200201	P2200210	P2200211
Two procedures in one visit, M and M, complexity level 3 and level 1	P2200300	P2200301	P2200310	P2200311
Two procedures in one visit, M and M, complexity level 2 and level 2	P2200400	P2200401	P2200410	P2200411
Two procedures in one visit, M and M, complexity level 2 and level 1	P2200500	P2200501	P2200510	P2200511
Two procedures in one visit, M and M, complexity level 1 and level 1	P2200600	P2200601	P2200610	P2200611
Three procedures in one visit, D, D, and D, complexity level 2 and level 2 and level 1	P3030800	P3030801	P3030810	P3030811
Three procedures in one visit, D, D, and D, complexity level 2 and level 1 and level 1	P3030900	P3030901	P3030910	P3030911
Three procedures in one visit, D, D, and D, complexity level 1 and level 1 and level 1	P3031000	P3031001	P3031010	P3031011
Three procedures in one visit, M, D, and D, complexity level 2 and level 2 and level 2	P3121000	P3121001	P3121010	P3121011
Three procedures in one visit, M, D, and D, complexity level 2 and level 1 and level 1	P3121200	P3121201	P3121210	P3121211
Three procedures in one visit, M, D, and D, complexity level 1 and level 2 and level 1	P3121700	P3121701	P3121710	P3121711
Three procedures in one visit, M, D, and D, complexity level 1 and level 1 and level 1	P3121800	P3121801	P3121810	P3121811
Three procedures in one visit, M, M, and D, complexity level 2 and level 1 and level 1	P3211500	P3211501	P3211510	P3211511
Three procedures in one visit, M, M, and D, complexity level 1 and level 1 and level 1	P3211800	P3211801	P3211810	P3211811
Three procedures in one visit, M, M, and M, complexity level 3 and level 2 and level 2	P3300400	P3300401	P3300410	P3300411
Three procedures in one visit, M, M, and M, complexity level 3 and level 2 and level 1	P3300500	P3300501	P3300510	P3300511
Three procedures in one visit, M, M, and M, complexity level 3 and level 1 and level 1	P3300600	P3300601	P3300610	P3300611
Three procedures in one visit, M, M, and M, complexity level 2 and level 2 and level 1	P3300800	P3300801	P3300810	P3300811
Three procedures in one visit, M, M, and M, complexity level 2 and level 1 and level 1	P3300900	P3300901	P3300910	P3300911
Three procedures in one visit, M, M, and M, complexity level 1 and level 1 and level 1	P3301000	P3301001	P3301010	P3301011
Four procedures in one visit, D, D, D, and D, complexity level 2 and level 2 and level 1 and level 1	P4041300	P4041301	P4041310	P4041311
Four procedures in one visit, D, D, D, and D, complexity level 2 and level 1 and level 1 and level 1	P4041400	P4041401	P4041410	P4041411
Four procedures in one visit, D, D, D, and D, complexity level 1 and level 1 and level 1 and level 1	P4041500	P4041501	P4041510	P4041511
Four procedures in one visit, M, D, D, and D, complexity level 2 and level 1 and level 1 and level 1	P4132000	P4132001	P4132010	P4132011
Four procedures in one visit, M, D, D, and D, complexity level 1 and level 2 and level 1 and level 1	P4132900	P4132901	P4132910	P4132911
Four procedures in one visit, M, D, D, and D, complexity level 1 and level 1 and level 1 and level 1	P4133000	P4133001	P4133010	P4133011
Four procedures in one visit, M, M, D, and D, complexity level 2 and level 1 and level 1 and level 1	P4223000	P4223001	P4223010	P4223011
Four procedures in one visit, M, M, D, and D, complexity level 1 and level 1 and level 1 and level 1	P4223600	P4223601	P4223610	P4223611

M = oral and maxillofacial surgery (OMFS), D = tooth and periodontium, P = multiple procedures in one visit, GA = general anesthesia, CC= complication and comorbidity.
